# Supplementary material for: Evidence supported by Mendelian randomization: impact on inflammatory factors in knee osteoarthritis
Source: Front Med (Lausanne). 2024 May 28;11:1382836. doi: 10.3389/fmed.2024.1382836 (PMC11165061; doi:10.3389/fmed.2024.1382836)
Supplement: Supplementary file 1 [file Data_Sheet_1.ZIP › data/GCST90274812_5e6/812.funnel_plot.pdf]

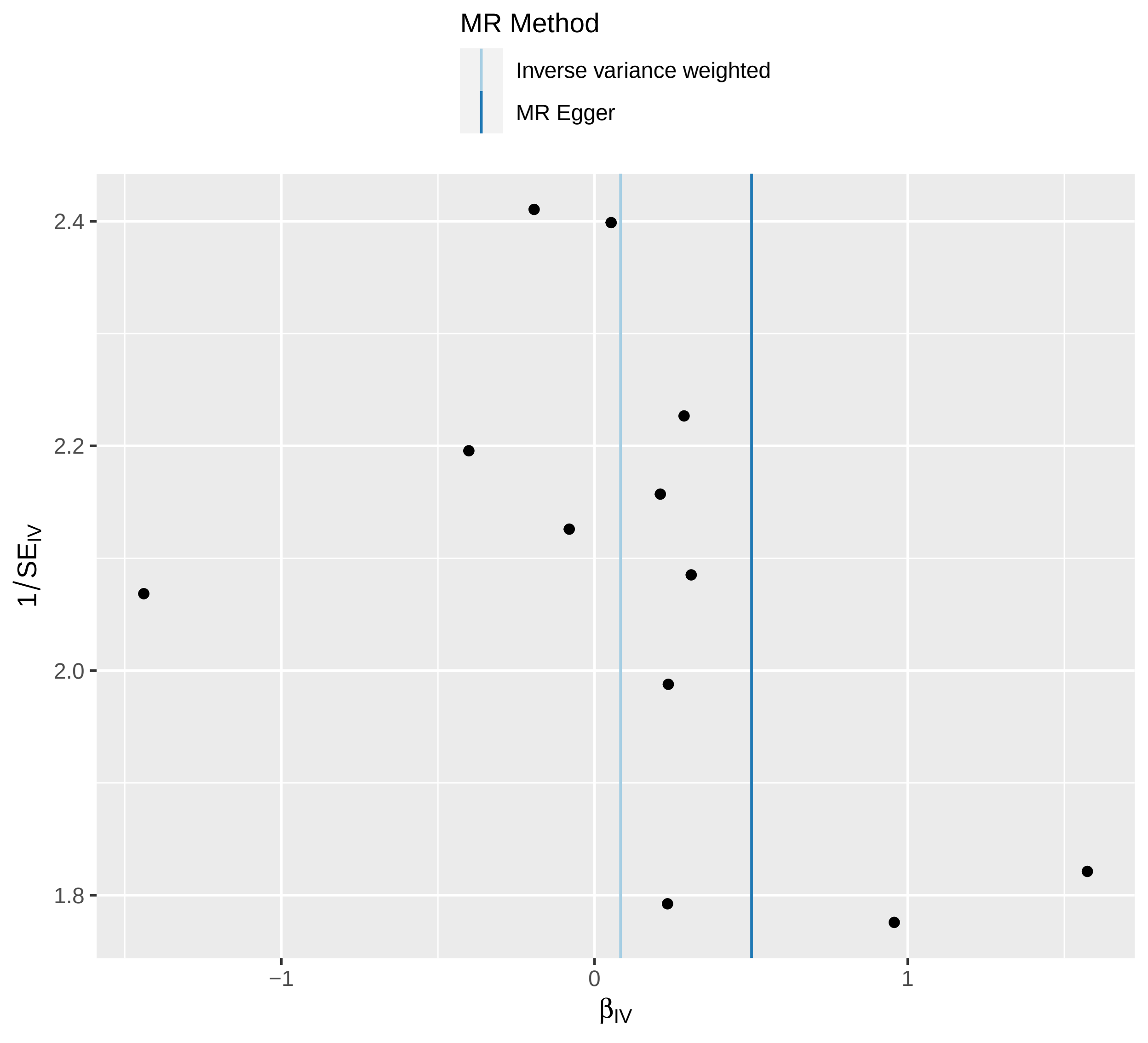
MR Method
Inverse variance weighted
MR Egger
2.4
2.2
2.0
1.8
1/SE_IV
−1
0
1
β_IV
